# Association between Leukoaraiosis and Symptomatic Intracranial Large Artery Stenoses and Occlusions: the Chinese Intracranial Atherosclerosis (CICAS) Study

**DOI:** 10.14336/AD.2018.0118

**Published:** 2018-12-04

**Authors:** Wanying Duan, Yuehua Pu, Haiyan Liu, Jing Jing, Yuesong Pan, Xinying Zou, Yilong Wang, Xingquan Zhao, Chunxue Wang, Yongjun Wang, Ka Sing Lawrence Wong, Ling Wei, Liping Liu, on behalf of the CICAS StudyGroup

**Affiliations:** ^1^Department of Neurology, Beijing Tiantan Hospital, Capital Medical University, Beijing, China.; ^2^China National Clinical Research Center for Neurological Diseases, Beijing, China.; ^3^Center of Stroke, Beijing Institute for Brain Disorders, China.; ^4^Beijing Key Laboratory of Translational Medicine for Cerebrovascular Disease, Beijing, China.; ^5^Department of Neurology, The Second Affiliated Hospital of Xuzhou Medical University, Xuzhou, China.; ^6^Department of Epidemiology and Health Statistics, School of Public Health, Capital Medical University, Beijing, China.; ^7^Department of Medicine and Therapeutics, the Chinese University of Hong Kong, Prince of Wales Hospital, Shatin, Hong Kong Special Administrative Region, China.; ^8^Department of Anesthesiology, Emory University School of Medicine, Atlanta, GA, USA.; ^9^Department of Neurology, Emory University School of Medicine, Atlanta, GA, USA.

**Keywords:** leukoaraiosis, intracranial arteriosclerosis, magnetic resonance imaging, ischemia

## Abstract

Leukoaraiosis (LA) is frequently found in ischemic stroke patients, especially when those patients have intracranial atherosclerosis (ICAS). However, previous studies regarding an association of LA with cerebral large artery atherosclerosis showed conflicting results, and the relationship of LA with ICAS is uncertain. This study aimed to explore the association between LA and cerebral large artery atherosclerosis in Chinese patients with cerebral ischemia. Data were derived from the Chinese Intracranial Atherosclerosis (CICAS) study. Patients diagnosed with an ischemic stroke or transient ischemic attack (TIA) within 7 days of symptom onset were included. The analysis of magnetic resonance imaging (MRI) focused on severity of LA in periventricular and deep white matter; type of cerebral large artery stenosis; and the number, severity, and distribution of ICAS lesions. ICAS was defined as an occlusion or more than 50% stenosis of intracranial vessels on magnetic resonance angiography. Among 2420 patients included, distinct LA was observed in 898 (37.11%) patients, and the rate of LA increased significantly with an increased number of risk factors. Multivariate analysis revealed that LA was independently associated with ICAS (odds ratio [OR], 1.388; 95% confidence interval [CI], 1.132-1.702; *P*=0.0016). In the subgroup analysis of ICAS, LA was more frequently observed in multiple lesions (OR, 1.342; 95% CI, 1.060-1.699; *P*=0.0146), occlusive lesions (OR, 1.554; 95% CI, 1.214-1.998; *P*=0.0005), and lesions in the posterior circulation (OR, 1.360; 95% CI, 1.003-1.846; *P*=0.0481). In this nationwide prospective study, LA was associated with symptomatic ICAS, patients with multiple ICAS lesions, occlusive lesions, and atherosclerotic lesions in the posterior circulation were more likely to coexist with LA.

Leukoaraiosis (LA) results in white matter lesions that are frequently seen as bilateral patchy or diffuse hyperintensities on T2-weighted and fluid-attenuated inversion recovery (FLAIR) sequences of magnetic resonance imaging (MRI). Pathological findings in regions of LA include demyelination, gliosis, lipohyalinosis, and fibrinoid necrosis [1]. LA is more common in elderly people, as well as in patients with known vascular risk factors and symptomatic cerebrovascular disease [2, 3].

Previous studies have indicated that LA is associated with progression of cognitive impairment, gait abnormalities, poor functional outcome, increased mortality, higher risk of hemorrhage after thrombolysis, and recurrence of ischemic stroke [4-7]. However, the pathogenesis of LA is still not completely understood. Arteriolosclerosis is considered to play an important role in the development of LA [1]. Although previous studies showed that LA was strongly related to cerebral small vessel disease [8-10], conflicting findings regarding the association between LA and large artery atherosclerotic diseases have been reported. Additionally, since most previous studies on the relationship between LA and cerebral artery stenosis have focused on extracranial carotid arteries, the evidence of association between LA and intracranial stenosis is limited and inconclusive [11, 12].

Therefore, in this nationwide, multicenter, prospective clinical study, we attempted to assess the association between intracranial atherosclerosis (ICAS) and LA in patients with ischemic stroke or transient ischemic attack (TIA) using MRI.

## MATERIALS AND METHODS

### Study Participants

Data were prospectively collected from the Chinese Intracranial Atherosclerosis (CICAS) study. The CICAS study is a nationwide, multicenter, government-funded, hospital-based, cohort study of data from consecutive patients with ischemic stroke or TIA. Twenty-two general hospitals covering a wide geographic area in China participated in this study. Details on the rationale, design and methodology of the CICAS study have been published previously [13]. The study was approved by the ethics committee of Beijing Tiantan Hospital and all participating hospitals. Informed consent was obtained from all recruited patients or their legal guardians before data collection.

Patients were enrolled in the study if they had an onset of symptoms <7 days and were aged between 18 and 80 years. The exclusion criteria were as follows: (1) patients who were clinically unstable, required close monitoring, were moribund, were disabled before admission (modified Rankin Scale score >2), were physically or subjectively unable to comply with magnetic resonance (MR) examination, or who had severe comorbidity; (2) patients with a known source of cardioembolism (including history of atrial fibrillation, valvular heart disease, or cardiac valve replacement) or who had atrial fibrillation and/or atrial flutter or valvular heart disease diagnosed by electrocardiography/ transesophageal, echocardiography/transthoracic, or echocardiography/Holter echocardiography during hospitalization, to avoid the inclusion of patients with vascular occlusion caused by cardioembolism; and (3) patients without available MR images identifying lesions and/or severity of LA. On admission, the information collected included age, sex, vascular risk factors (hypertension, diabetes mellitus, hyperlipidemia, heart disease, previous stroke, current smoker, and heavy drinker), medical history, and physical examination [13]. The definitions of the main vascular risk factors were detailed in prior studies [13, 14]. All patients underwent specified clinical evaluation, including laboratory tests, 12-lead electrocardiography, brain MRI, 3-dimensional (3D) time-of-flight (TOF) MR angiography (MRA) for cerebral circulation, and duplex color Doppler ultrasound or contrast-enhanced MRA for extracranial carotid vessel evaluation.

From October 2007 to June 2009, a total of 2864 patients with noncardioembolic ischemic cerebrovascular disease was consecutively recruited into CICAS. Among them, 436 patients were not included in further analysis because the quality of MRI was not sufficient to reliably assess the presence and severity of LA, and 8 patients were excluded due to incomplete baseline data. Finally, our study consisted of 2420 patients for analysis.

### Image Analysis/Interpretation 

All patients underwent conventional MRI and 3D TOF MRA on a 3.0- or 1.5-T MR scanner within 3 days of admission. The acquisition parameters of MRI consisted of T1-weighted imaging (repetition time [TR]/echo time [TE], 1200/11 ms), T2-weighted imaging (TR/TE, 4500/84 ms), FLAIR (TR/TE, 7000/94 ms; inversion time, 2500 ms), T2*-weighted gradient recalled echo (GRE) imaging (TR/TE, 613/20 ms), and diffusion-weighted imaging (DWI; TR/TE, 3000/75 ms). All above sequences utilized a 5-mm slice thickness and 1.5-mm interslice gap. Three-dimensional TOF MRA was also performed (TR/TE, 20-25/3.3-3.9 ms for the 3.0-T scanner, 25-35/6.9 ms for the 1.5-T scanner; flip angle, 15º-20º for the 3.0-T scanner, 25º-35º for the 1.5-T scanner; slice thickness=0.65-1.00 mm).

LA was defined as a hyperintense lesion on both T2-weighted imaging and FLAIR, but was usually not seen on T1-weighted imaging or showed faint hypointensity [15]. DWI was used to differentiate acute ischemic stroke lesions from LA. We rated LA using the Manolio method to estimate the overall volume of periventricular and subcortical white matter signal abnormality, and the severity of LA was classified based on a scale from 0 to 9 [7, 16, 17]. We classified patients with LA of grade 3 or higher as having LA and of grade 2 or lower as being non-LA [7, 17].

Lacune were defined as lesions 3-15 mm in size with the same signal characteristics as cerebrospinal fluid on all sequences, with a hyperintense rim on the FLAIR sequence [15], and were differentiated from dilated Virchow-Robin spaces by their wedge shape and surrounding hyperintensity on FLAIR [14, 18]. Microbleeds were defined as focal, small (<10 mm in diameter), homogeneous, punctate, rounded or oval lesions of low signal on T2*-weighted GRE MRI; symmetrical lesions in the globus pallidus (likely representing calcification or iron deposition); hypointense lesions in the subarachnoid space (likely representing adjacent pial blood vessels) were excluded [19]. A complete circle of Willis was considered to include the anterior communicating artery, bilateral A1, terminal internal carotid artery, P1, and posterior communicating artery; if any segment of the circle of Willis was missing, it was considered incomplete [13].

**Table 1:** Baseline Characteristics of Participants.

Characteristics	Total (*n*=2420)	Without LA (*n*=1522)	With LA (*n*=898)	*p* value
Age*, y	61.9±11.2	59.0±11.1	67.2±9.2	<0.0001
Male sex	1634 (67.52)	1037 (68.13)	597 (66.48)	0.4021
Hypertension	1884 (77.85)	1115 (73.26)	769 (85.63)	<0.0001
Diabetes mellitus	858 (35.45)	522 (34.30)	336 (37.42)	0.1218
Hyperlipidemia	1841 (76.07)	1179 (77.46)	662 (73.72)	0.0378
Heart disease	228 (7.96)	145 (7.38)	83 (9.24)	0.0906
Peripheral vascular disease	17 (0.70)	14 (0.92)	3 (0.33)	0.0779
Family history of stroke	250 (10.33)	166 (10.91)	84 (9.35)	0.2225
History of cerebral ischemia	1745 (72.11)	957 (62.88)	788 (87.75)	<0.0001
History of hemorrhage stroke	43 (1.78)	11 (0.72)	32 (3.56)	<0.0001
Current smoker	854 (35.29)	601 (39.49)	253 (28.17)	<0.0001
Heavy drinker	113 (4.67)	88 (5.78)	25 (2.78)	0.0005
Multiple infarctions	260 (14.12)	171 (15.00)	89 (12.68)	0.1620
Pattern of infarct				0.0036
No infarct	552 (23.07)	368 (24.42)	184 (20.77)	
Supratentorial	1382 (57.76)	855 (56.73)	527 (59.48)	
Infratentorial	423 (17.68)	264 (17.52)	159 (17.95)	
Supratentorial and infratentorial	36 (1.50)	20 (1.33)	16 (1.81)	
Lacune	980 (40.50)	410 (26.94)	570 (63.47)	<0.0001
Cerebral microbleeds	192 (7.93)	64 (4.20)	128 (14.25)	<0.0001
Complete circle of Willis	120 (4.96)	89 (5.85)	31 (3.45)	0.0071

LA indicates leukoaraiosis.

* Continuous variables with normal distribution expressed as mean ± mean±standard (SD); other values are expressed as n (%).

The intracranial arterial segments we assessed included the bilateral intracranial internal carotid artery (an intracranial location was defined as distal to the ophthalmic artery), anterior cerebral artery A1/A2, middle cerebral artery M1/M2, posterior cerebral artery P1/P2, distal vertebral artery (including the intradural V4 segment), and basilar artery [13]. According to the severity of stenosis, we classified the patients into four groups using the published method for the Warfarin-Aspirin Symptomatic Intracranial Disease Study: <50% or no stenosis, 50%-69% stenosis, 70%-99% stenosis, and occlusion [20]. ICAS was defined as ≥50% diameter reduction on MRA. We estimated the extracranial portion of the internal carotid artery by contrast-enhanced MRA or ultrasonographic examination with the published diagnostic criteria [21]. According to the distribution of stenosis, we categorized vessels with >50% stenosis or occlusion into intracranial stenosis, extracranial stenosis, and mixed groups (both intracranial and extracranial stenosis) [13]. Furthermore, according to the distribution of ICAS, we categorized ICAS vessels into anterior circulation, posterior circulation, and mixed groups (both anterior circulation and posterior circulation) [22]. The posterior cerebral artery stenosis was considered as in the anterior circulation only when ICAS lesion was located in the full fetal origin of the posterior cerebral artery, uni- or bilateral, which the P1 segment was absent [23, 24].

All MRI and MRA images were stored in digital format and read independently by 2 radiologists (XY Zou and Y Soo) who were blinded to patients’ clinical information and outcomes. A third reader reviewed the measurements and decided between the two when the disagreements were greater than 10%.

### Statistical Analysis 

Categorical variables were summarized as frequency numbers. Continuous variables were described as median (interquartile range) or mean ± standard deviation (SD), as appropriate. For the univariate analysis, we used Pearson’s χ^2^ test or Fisher’s exact test for categorical variables and the Independent Samples *t*-test or Wilcoxon test for continuous variables. Normality was assessed using the Kolmogorov-Smirnov test. Variables with a *p*-value <0.05 as calculated by univariate analysis were included in the multiple logistic regression analysis. All data were analyzed with SAS version 9.4 software (SAS Institute Inc., Cary, NC, USA). The level of statistical significance was set at *P*<0.05.

**Table 2:** Independent Association Between Leukoaraiosis and Baseline Characteristics.

	OR (95% CI)	*p* Value
Age	1.075 (1.064-1.086)	<0.0001
Hypertension	1.316 (1.018-1.702)	0.0361
Hyperlipidemia	0.877 (0.700-1.100)	0.2570
History of cerebral ischemic	2.224 (1.704-2.902)	<0.0001
History of hemorrhage stroke	5.072 (2.333-11.028)	<0.0001
Current smoker	0.862 (0.690-1.077)	0.1918
Heavy drinker	0.633 (0.371-1.082)	0.0947
Infratentorial infarct pattern	1.505 (1.160-1.953)	0.0021
Lacune	3.154 (2.555-3.895)	<0.0001
Cerebral microbleeds	2.537 (1.776-3.625)	<0.0001
Complete circle of Willis	0.867 (0.538-1.396)	0.5567

OR indicates odds ratio; 95% CI, 95% confidence interval.

### RESULTS

Of 2420 participants included in these analyses, the mean age was 61.9±11.2 years (range, 19-80 years), and 1634 (67.52%) were men. Among these, distinct LA was observed in 898 (37.11%) patients. Baseline characteristics of all patients are summarized in Table 1. Patients with LA had older age at onset of cerebral ischemia disease than did patients without LA. In terms of vascular risk factors, patients with LA had a higher rate of hypertension, history of cerebral ischemia, and history of hemorrhage stroke than did non-LA patients, but had a lower rate of hyperlipidemia, current smoking, and heavy drinking. Of 1868 (77.19%) acute ischemic stroke events, LA was more prevalent in patients whose infarct lesions were supratentorial (59.48%), infratentorial (17.95%), or mixed supratentorial and infratentorial (1.81%). In addition, patients with LA had more lacune (63.47%) and cerebral microbleeds (14.25%). Meanwhile, the prevalence of LA was significantly lower for patients who had a complete circle of Willis. On multivariable logistic regression analysis, age, hypertension, history of cerebral ischemia, history of hemorrhage stroke, infratentorial infarct pattern, lacune, and cerebral microbleeds were found to be independently associated with LA (Table 2).

**Table 3:** ICAS Characteristics of Patients with and without LA.

	Total (*n*=2420)	Without LA (*n*=1522)	With LA (*n*=898)	*p* value
ICAS	1155 (47.73)	675 (44.35)	480 (53.45)	<0.0001
Distribution of stenosis				0.0002
None	1146 (47.36)	765 (50.26)	381 (42.43)	
Intracranial only	930 (38.43)	549 (36.07)	381 (42.43)	
Extracranial only	119 (4.92)	82 (5.39)	37 (4.12)	
Intracranial and extracranial	225 (9.30)	126 (8.28)	99 (11.02)	
Multiple ICAS*	517 (21.36)	283 (18.59)	234 (26.06)	<0.0001
Severity of ICAS†				0.0007
None or <50%	1265 (52.27)	847 (55.65)	418 (46.55)	
50% to 69%	290 (11.98)	163 (10.71)	127 (14.14)	
70% to 99%	218 (9.01)	129 (8.48)	89 (9.91)	
100%	647 (26.74)	383 (25.16)	264 (29.40)	
Distribution of ICAS‡				<0.0001
None	1265 (52.27)	847 (55.65)	418 (46.55)	
Anterior	569 (23.51)	376 (24.70)	193 (21.49)	
Posterior	289 (11.94)	156 (10.25)	133 (14.81)	
Anterior and posterior	297 (12.27)	143 (9.40)	154 (17.15)	

Values are expressed as n (%). ICAS indicates intracranial atherosclerosis; LA, leukoaraiosis.

**Figure 1.**
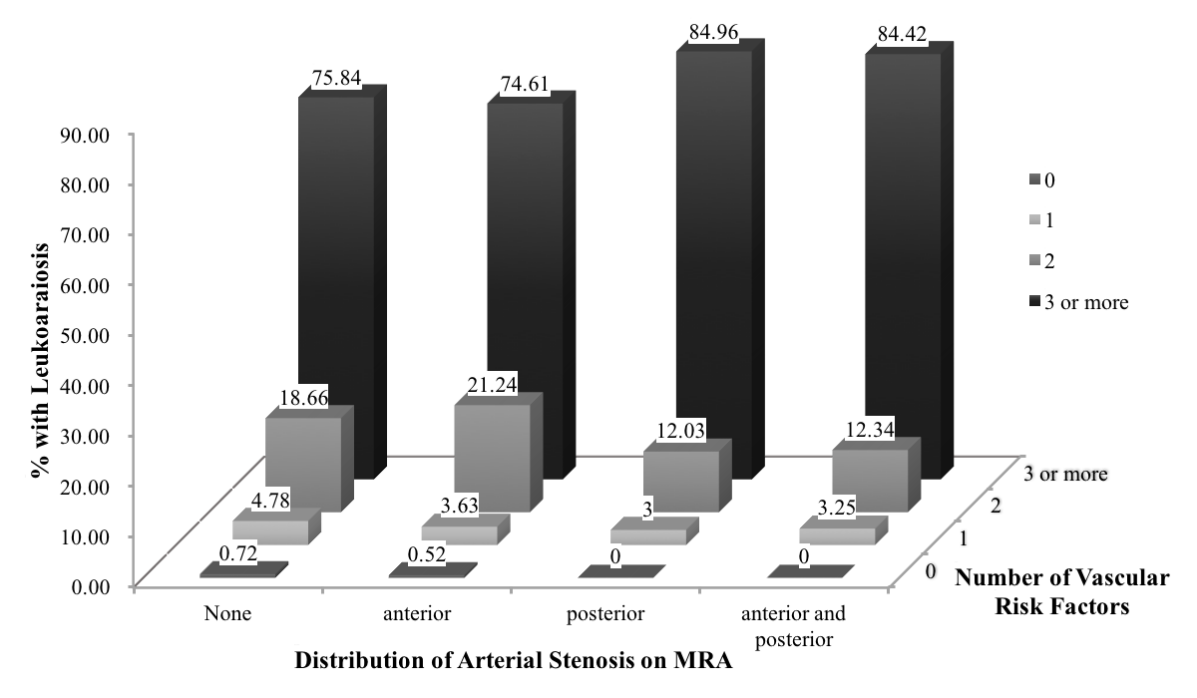
Percentage of patients with leukoaraiosis according to the distribution of stenosis and number of vascular risk factors. The number above each block indicates the percentage of patients with leukoaraiosis. MRA, magnetic resonance angiography.

In total, the prevalence of ICAS was 47.73% (1155 patients, including 225 patients with coexisting extracranial carotid stenoses or occlusions). The ICAS characteristics of patients with and without LA are presented in Table 3. Among patients with ICAS, patients with LA were more likely than non-LA patients to have intracranial atherosclerotic lesions (53.45% versus 44.35%; *P*<0.0001)—both intracranial only (42.43% versus 36.07%; *P*=0.0002) and combined intracranial and extracranial lesions (11.02% versus 8.28%; *P*=0.0002). Among patients with LA, 127 patients (14.14%) had 50%-69% stenosis, 89 patients (9.91%) had 70%-99% stenosis, and 264 patients (29.40%) had occlusion. Patients with LA had significantly higher rates of multiple ICAS lesions (26.06% versus 18.59%; *P*<0.0001) than did patients without LA. Intracranial atherosclerotic lesions in the posterior circulation were more common in patients with LA than in patients without LA, whether in the posterior circulation only (14.81% versus 10.25%; *P*<0.0001) or both anterior and posterior circulation (17.15% versus 9.40%; *P*<0.0001). Since LA had a high burden of vascular risk factors (including age, hypertension, and history of stroke), we further evaluated their relationship by the distribution of stenosis and number of risk factors. The proportion of LA showed a significant rising trend with the increased number of risk factors (Fig. 1). Furthermore, the highest rate of LA was observed in patients with posterior circulation ICAS and the presence of ≥3 vascular risk factors.

In the multivariable logistic analysis adjusted for all potential confounding variables, the presence of LA was independently associated with ICAS. Furthermore, the multivariable logistic analysis of subgroups revealed that multiple intracranial atherosclerotic lesions, intracranial large artery occlusion, and posterior circulation ICAS were independently associated with the development of LA (Table 4).

## DISCUSSION

The burden of stroke in China has increased over the past 30 years, and is likely the highest in the world [13, 25, 26]. Previous studies have revealed that there is a high prevalence of intracranial atherosclerotic stenosis in patients with stroke or TIA in Asia [13, 17, 27, 28], and the proportion of ICAS in our study accounted for 47.73%. Our data indicated that LA was common in patients with symptomatic ICAS in China and was associated with intracranial large artery stenotic and occlusive diseases. Moreover, we further confirmed that patients with multiple ICAS lesions, occlusive lesions, and atherosclerotic lesions in the posterior circulation may be particularly prone to have coexistent LA.

Although the pathophysiology of LA is not well understood, there is evidence to suggest that LA may be linked to cerebrovascular disease and atherosclerosis [2]. In the past two decades, there is increasing evidence about the relationship between LA and large-artery atherosclerosis. However, researches on the relationship between LA and cerebral artery stenosis have focused on extracranial carotid arteries and the findings were inconclusive [29]. Some studies suggested that LA was associated with carotid atherosclerosis [30-32], whereas others shown an inverse or no correlation [29, 33, 34]. With the development of the modern technology of neuroimaging, people are increasingly interested in intracranial large artery disease. It has been previously reported that LA is more prevalent in the large artery atherosclerosis stroke subtype among Korean patients with ischemic stroke and is associated with ICAS rather than with extracranial atherosclerosis [17, 29]. In addition, a recent retrospective study demonstrated that ICAS is associated with the volume of white matter hyperintensity in a healthy population [12]. Although a single-center, small-sample study suggested that LA had no relationship with cerebral large artery stenosis, multiple intracranial large artery stenosis was found to increase the risk of LA [35]. However, most of the previous studies are often limited by being single-center series, small sample size, and from western countries. As the first large, prospective, multicenter, cohort study to provide comprehensive data about the distribution and prognosis of ICAS in Asia, CICAS offers us an opportunity to explore the relationship between LA and symptomatic ICAS in China. In our study, we found that LA was related not only to the presence of ICAS but also to the number and severity of intracranial large artery atherosclerotic lesions. Although the pathogenesis of LA is still unclear, there is evidence to suggest that LA may have multiple etiologies [36]. Ischemia is thought to be the most important etiology [36, 37]. Several previous studies demonstrated that cerebral perfusion and cerebrovascular reactivity are reduced in areas with LA, which was attributed to the vulnerable nature of the long penetrating end-arteries that feed the white matter [38]. It seems that not focal ischemia such as small vessel stroke, but diffuse ischemia from large arterial blood supply insufficiency is responsible for the progression of LA. Occlusion of intracranial large arteries could reduce brain perfusion, diminish cerebral vasomotor reactivity, and induce chronic cerebral ischemia, and the chronic hypoperfusion state could accelerate lipohyalinosis [12, 38].

**Table 4:** Association Between Leukoaraiosis and ICAS.

	Crude OR (95% CI)	*p* value	Adjusted^a^ OR (95% CI)	*p* value
ICAS	1.441 (1.221-1.700)	<0.0001	1.388 (1.132-1.702)	0.0016
Distribution of stenosis				
None	1			
Intracranial only	1.393 (1.165-1.667)	0.0003	1.320 (1.060-1.644)	0.0132
Extracranial only	0.906 (0.603-1.361)	0.6347	-	-
Intracranial and extracranial	1.578 (1.180-2.109)	0.0021	1.405 (0.988-1.998)	0.0581
Multiple ICAS*	1.543 (1.267-1.879)	<0.0001	1.342 (1.060-1.699)	0.0146
Severity of ICAS†				
None or <50%	1			
50% to 69%	1.579 (1.217-2.047)	0.0006	1.308 (0.959-1.783)	0.0898
70% to 99%	1.398 (1.041-1.877)	0.0257	1.136 (0.805-1.603)	0.4690
100%	1.397 (1.148-1.699)	0.0008	1.554 (1.214-1.998)	0.0005
Distribution of ICAS‡				
None	1			
Anterior	1.040 (0.844-1.282)	0.7129	-	-
Posterior	1.728 (1.333-2.239)	<0.0001	1.360 (1.003-1.846)	0.0481
Anterior and posterior	2.182 (1.689-2.819)	<0.0001	1.769 (1.304-2.400)	0.0002

ICAS indicates intracranial atherosclerosis; OR, odds ratio; 95% CI, 95% confidence interval. Adjusted^a^: adjusted for age, sex, hypertension, Hyperlipidemia, history of cerebral ischemic, history of hemorrhage stroke, current smoker, heavy drinker, pattern of infarct, lacune, cerebral microbleeds, and complete circle of Willis.

* ≥2 stenoses in any arterial segments.

† Rate of stenosis. If stenosis was multiple, the more serious stenosis was recorded.

‡ Posterior: stenosis in posterior circulation; anterior and posterior: stenosis in both anterior and posterior circulation.

In our study, we demonstrated that LA was independently associated with posterior circulation ICAS, which had not been shown before. In addition, our study found that patients with infratentorial infarctions had a 1.505-fold risk of coexisting with LA. Previous necropsy studies have shown that intracranial occlusive disease is more common in the posterior circulation [39], especially in the intracranial vertebral arteries and basilar artery [40, 41]. Furthermore, studies of infratentorial infarctions revealed that patients with cerebellar (especially in posterior inferior cerebellar arteries regions), lateral medullary, or hemimedullary infarcts have a very high frequency of occlusive intracranial vertebral artery lesions [42-44]. Basilar artery occlusions were commonly observed in patients with paramedian pontine and midbrain infarcts [45, 46]. In the Lausanne Stroke Registry, more than half of patients with branch territory distribution infarcts in the posterior circulation had large artery occlusive lesions on MRA that could have obstructed branches [47]. Moreover, the posterior circulation consists of relatively more brain tissue in the brainstem and thalamus supplied by small penetrating arteries than does the anterior circulation [48]. Our results suggested that intracranial large artery atherosclerotic lesions located in the posterior circulation, particularly with multiple ICAS lesions, may be more vulnerable to hemodynamic insufficiency, leading to the formation of LA. Additionally, posterior circulation ICAS may also result in occlusion of the large vessels branch or penetrating branch in its intraparenchymatous course, which may reduce blood supply and enlarge LA lesions.

Our results showed that LA and atherosclerotic disease have some risk factors in common and may, therefore, occur concurrently; these include age, hypertension, and history of stroke. Further analysis showed that the rate of LA increased as the number of vascular risk factors increased, and it increased when accompanied by ICAS. These findings suggested that vascular risk factors may be a pathophysiologic connector between ICAS and LA, and provided strong evidence of a vascular basis for LA. In previous studies, increasing age and hypertension are the only consistently identified and generally accepted risk factors for LA [17, 49]. Alteration of blood supply to the white matter due to arteriosclerosis during the aging process may be a possible reason for the strong association between age and LA [1, 50]. Hypertension is also strongly associated with white matter disease and is probably the most important modifiable risk factor [50]. Hypertension may induce lipohyalinosis and fibrinoid necrosis of small perforating arteries, which can further lead to chronic ischemia and blood-brain barrier breakdown [1]. Thus, it is possible that control of vascular risk factors can prevent progression of LA; further randomized controlled trials are necessary to prove this.

Strengths of our study include the following: (1) the results are from a multicenter, large-sample, consecutive cohort study that used MRA as the screening tool for ICAS; (2) the prospective inclusion of patients, controls in multiple centers using an identical protocol, and standardized forms across all centers; (3) MRI-based rating of leukoaraiosis burden; (4) blinded assessment of leukoaraiosis and other imaging information with respect to clinical variables; and (5) adjustment for clinically relevant confounders. Above all, these factors contributed to decreased diagnostic errors for LA and ICAS.

Our study has several limitations. First, although the results were based on a prospective cohort study, analysis of LA and ICAS was cross-sectional, limiting our ability to assess the longitudinal effect of large artery atherosclerosis on white matter changes. Second, our study was hospital based, and those who did not meet the inclusion criteria were excluded; some degree of selection bias existed. Additionally, the visual semi quantitative analysis of LA via a 0-to-9 grading system may not reflect an accurate estimation of the LA severity, although it has been shown to correlate well with the leukoaraiosis volume [51] and has demonstrated high interrater reliability of leukoaraiosis assessment [16]. Additional quantitative grading systems, including volumetric methods for LA measurement, are needed.

### Conclusions

In conclusion, our study demonstrates that LA is independently associated with symptomatic ICAS, patients who have multiple ICAS lesions, occlusive lesions, and atherosclerotic lesions in the posterior circulation are at particularly high risk of coexistent LA. Identification of patients at high risk and strict control of vascular risk factors may be helpful in preventing the development of LA in clinical practice. Further studies are needed to validate our findings and explore their clinical implications.
